# The Festival Season

**Published:** 1892-03-19

**Authors:** 


### PASSING TOPICS.

### THE FESTIVAL SEASON.

The advertising columns of the daily papers afford ample evidence of the return of the festival season, and the supporters of hospitals and other institutions are being plied with the customary invitations either to dine, donate, or do both. Let us hope the results will be commensurate with the trouble involved and gratifying all round, not least to those who dine. We are led to utter this pious wish, because the announcement made in the *Standard* when recording the last Festival of the Royal Caledonian Asylum, that "as a result of the dinner the secretary announced annual subscriptions and donations amounting to close upon £2,000, including a legacy of £1,000," seems alarming, and we trust it was not intended to be taken literally, else we might certainly apprehend a serious falling off in the attendance at the charitable board, for to donate, dine, and die in one evening would be too unhappy a conjunction of circumstances even for the most earnest and self-subordinating multiplier of social engagements.

The world at large, a conveniently concise phrase for describing everybody who is ignorant or negligent of the subject under notice, has little or no conception of the heroic labours incidental to the conduct of a charitable campaign, more especially when, as in the case of a festival, there is so all important a preliminary undertaking as catching a chairman. It is true that when caught he may sometimes prove rather more ornamental than useful, but none the less, in one form or the other, he is indispensable, and the ultimate capture represents an amount of work and waiting which only those who have taken part in the chase can fully realise. Society in general may occasionally note that such and such an illustrious personage has consented to preside at this or that "function," but it little recks all the announcement means. Those solicited are not to be blamed. The few highly-placed men who are known to be willing to give their assistance in this form are overwhelmed with applications. Their position is exactly analogous to that of the subscribers: they have not only their own share of duty to perform, they must make up for other people's deficiencies as well.

What we think open to criticism is the growing disposition on the part of chairmen and stewards, especially the latter, to consider the office purely honorary and without any definite duties. Names however eminent will not of themselves ensure a tangible result. If the stewards of a festival do not bestir themselves but leave all the collecting to the hospital officials, we do not see how the institution is really advantaged by the proceeding. Dummy stewards might well be allocated to a dummy dinner, and the expense of dining, a very considerable item, would be saved. Charities generally want in this matter to learn something of the methods of Freemasons. Their charitable festivals, we believe, are almost always conspicuous successes, and for the simple reason that they are not content merely to lend their names, nor to dine only, nor even to give, but they co-operate in the fullest sense of the term by utilising what opportunities they possess to bring in fresh supporters. It is only when such views prevail that festivals can be made the means of compassing great ends. Drones, of course, will congregate about every hive, but if we want honey we must have working bees.

Agricola.

